# Adolescent Dietary Supplementation of Omega‐3 Polyunsaturated Fatty Acids Alleviates PTSD‐Like Fear Memory and Affective Dysregulation in Rats

**DOI:** 10.1002/smmd.70049

**Published:** 2026-08-04

**Authors:** Wenjing Li, Yi Yin, Jie Li, Ziwei Yao, Siqi Wang, Yinan Jin, Lihua Bian, Jianyou Guo

**Affiliations:** ^1^ State Key Laboratory of Cognitive Science and Mental Health Institute of Psychology Chinese Academy of Sciences Beijing China; ^2^ Department of Psychology University of Chinese Academy of Sciences Beijing China; ^3^ COFCO Nutrition and Health Research Institute Co. Ltd. Beijing China

**Keywords:** gut microbiota, lipid metabolism, omega‐3 polyunsaturated fatty acids, PI3K/AKT/mTOR pathway, PTSD

## Abstract

Post‐traumatic stress disorder (PTSD) is a debilitating psychiatric disorder affecting approximately 3.9% of individuals worldwide over their lifetimes. Since adolescence is characterized by ongoing brain maturation and increased responsiveness to environmental stressors, this developmental stage may provide an important window for PTSD intervention. Growing evidence supports an association between ω‐3 polyunsaturated fatty acids (PUFAs), especially docosahexaenoic acid (DHA) and eicosapentaenoic acid (EPA), and the restoration of stress‐induced neural damage. However, how ω‐3 PUFA supplementation during adolescence affects adult susceptibility to PTSD remains poorly understood. Here, we observed significantly reduced DHA and EPA concentrations in the prefrontal cortex (PFC) and serum of PTSD‐susceptible rats. Providing 1.2% DHA and EPA in the adolescent diet significantly reduced PTSD‐like phenotypes in adulthood, including excessive fear memory retention, depressive‐like and anxiety‐like behaviors, while also reducing serum levels of proinflammatory cytokines. These phenotypic and inflammatory improvements were associated with changes in the gut microbiome, including its composition and diversity. DHA/EPA supplementation was also linked to alterations in multiple lipid species, with serum phosphatidylcholine levels showing a significant correlation with fear memory expression. Moreover, we observed dysregulation of the PI3K/AKT/mTOR pathway in the PFC of PTSD‐like rats, which was less pronounced in DHA/EPA‐supplemented animals. Collectively, these findings provide the first evidence relating adolescent ω‐3 PUFA status to PTSD‐like phenotypes in adulthood. It further identifies that gut microbiota composition, lipid metabolic profiles (notably phosphatidylcholine), and prefrontal PI3K/AKT/mTOR signaling are correlated with these behavioral outcomes, suggesting that these systems may collectively contribute to trauma resilience.

## Key Points

- Adolescent omega‐3 polyunsaturated fatty acids supplementation promotes resilience to trauma‐induced psychopathology in adulthood.
- PTSD susceptibility is associated with reduced prefrontal and circulating DHA/EPA availability.
- Omega‐3‐mediated behavioral protection coincides with restoration of gut microbial homeostasis.
- Phosphatidylcholine metabolism is closely linked to the expression of PTSD‐like fear memory.
- Recovery of prefrontal PI3K/AKT/mTOR signaling may contribute to omega‐3‐induced trauma resilience.

## Graphical abstract

Adolescent omega‐3 polyunsaturated fatty acid supplementation enhances resilience to trauma in adulthood. DHA/EPA intake restores gut microbial balance, reprograms lipid metabolism, and normalizes prefrontal PI3K/AKT/mTOR signaling. These changes may collectively mitigate PTSD‐like fear memory and affective dysregulation.

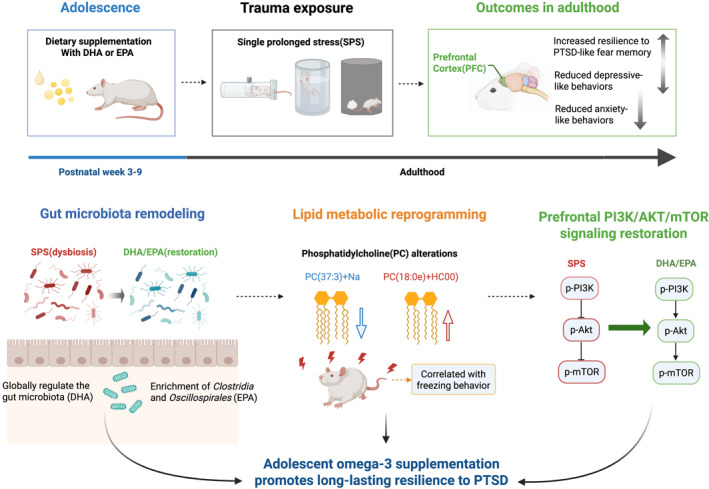

## 1 Introduction

As a chronic mental disorder, post‐traumatic stress disorder (PTSD) can occur after someone experiences something extremely threatening or catastrophic [1, 2]. It is estimated that nearly 70% of people worldwide experience a potentially traumatic event in their lifetime [3], but only about 5.6% eventually develop PTSD [4]. This indicates substantial interindividual differences in trauma susceptibility. Evidence suggests that trauma susceptibility is linked to many pathophysiological processes, including enhanced hypothalamic‐pituitary‐adrenal (HPA) axis activity [5, 6] and altered prefrontal‐limbic circuits during adolescence [7, 8]. Adolescence involves extensive neural rewiring and heightened sensitivity to stressors. This developmental window may therefore be especially critical in determining later susceptibility to stress‐related disorders [9, 10]. Thus, neurobiological support during this period may enhance stress adaptation and reduce the risk of PTSD in later life.

Omega‐3 polyunsaturated fatty acids (ω‐3 PUFAs) are crucial for brain development and central nervous system function [11, 12]. However, since humans cannot synthesize them, they rely largely on dietary intake [13]. Among these fatty acids, docosahexaenoic acid (DHA) and eicosapentaenoic acid (EPA) are considered the principal bioactive forms [14]. Once enter the bloodstream, they can reach the brain via passive diffusion [15] or transport proteins [16, 17]. Indeed, accumulating evidence suggests that DHA and EPA exert beneficial effects in stress‐related neuropsychiatric disorders. They are thought to act by relieving neuroinflammation and immune responses [18, 19], regulating monoaminergic signaling [20, 21], and protecting synaptic plasticity [22]. Clinical studies have further indicated that higher serum ω‐3 PUFA levels may be associated with lower PTSD risk [23]. These findings highlight the potential of ω‐3 PUFAs as nutritional interventions for stress‐related disorders.

Nevertheless, several key questions remain unanswered. Most animal studies so far have investigated ω‐3 supplementation only during adulthood, have focused mainly on limited behavioral outcomes such as memory impairment, and have not explored the underlying mechanisms. In other words, little attention has been given to developmental timing, affective symptoms, or integrated mechanistic pathways [24]. Recent longitudinal studies have reported associations between circulating unsaturated fatty acids and PTSD symptom trajectories [25]; however, these findings remain observational and cannot explore physiological mechanisms. Moreover, most experimental studies have used mixed ω‐3 formulations, leaving the potentially distinct contributions of DHA and EPA insufficiently characterized. Given that adolescence represents a sensitive developmental period, whether adolescent ω‐3 PUFA supplementation influences later susceptibility or resilience to traumatic stress remains to be determined.

PTSD and the gut microbiota seem to engage in a dynamic bidirectional crosstalk [26, 27]. Dietary lipids are increasingly viewed as key determinants that shape the composition and function of the gut microbial community [28]. Lipids not only provide energy but also influence the microbial ecology and host physiological processes [29]. Conversely, stress exposure can lead to microbiota dysbiosis [30, 31]. This often means a loss of some beneficial bacteria and an overgrowth of pro‐inflammatory microorganisms [32, 33]. The ensuing inflammation and metabolic disturbances may heighten susceptibility to stress‐related psychopathology [34]. Interestingly, as precursors of lipid‐derived signaling molecules, PUFAs play an important role in the regulation of metabolism and inflammation [27]. Therefore, modulating lipid metabolism and gut microbiota using PUFAs may represent a potential intervention approach for PTSD.

In this study, we evaluated how adolescent administration of DHA and EPA influences PTSD‐like behaviors in adulthood and explored the underlying mechanisms. We first analyzed the relationship between DHA/EPA levels in the prefrontal cortex (PFC) and serum and susceptibility to PTSD induced by single prolonged stress (SPS) in adult rats (Study 1). In Study 2, we gave juvenile rats DHA/EPA supplements continuously until adulthood. After that, we evaluated how this long‐term supplementation affected their PTSD‐like fear memory and emotional dysregulation. We also measured peripheral inflammatory cytokines, profiled the gut microbiota, and analyzed serum lipid profiles. Finally, we assessed the activation of relevant signaling pathways to demonstrate how dietary ω‐3 PUFA supplementation might affect PTSD‐like symptoms.

## 2 Methods

### 2.1 Animal

Rat adolescence generally starts around weaning (PND 21–28) and lasts until about PND 63, the age when they become sexually mature [35]. Animals in these experiments were SPF‐grade male Wistar rats obtained from *Charles River Laboratories.* We carried out two studies. Study 1 involved adult rats (PND 63, *n* = 48); Study 2 employed rats raised from juvenile stage (PND 21) to adulthood (*n* = 48: 16 for pilot experiment and 32 for formal experiment) with chronic DHA/EPA supplementation throughout development (Supporting Information S1: Table S1). Animals were maintained under a 12‐h light/dark cycle with *ad libitum* access to food and water.

### 2.2 Procedures and Administration

#### 2.2.1 Study 1

Random assignment of the 48 adult animals (PND 63) resulted in two groups: control group (*n* = 12) and SPS group (*n* = 36). Following a 7‐day environmental acclimation, all animals underwent the SPS procedure. To evaluate PTSD‐like behaviors, rats underwent a series of behavioral tests 14 days after SPS. In line with previous practices with certain modifications [36], the 36 rats in the SPS group were stratified based on the FCT freezing percentage by quartile analysis. Specifically, rats with a freezing percentage in the FCT falling below the 25th percentile or above the 75th percentile were designated as the resilience group (*n* = 9) or susceptibility group (*n* = 9), whereas those falling between these thresholds were designated as the normal response group (*n* = 18). We quantified DHA and EPA in the PFC and serum using LC‐MS (*n* = 3 per group, randomly selected from the control, susceptibility, and resilience groups), and subsequently analyzed their links to PTSD‐like behavioral phenotypes.

#### 2.2.2 Pilot Experiment of Study 2

Before the formal experiment of Study 2, we conducted a pilot experiment to evaluate the palatability of DHA‐ and EPA‐supplemented diets for juvenile rats. Sixteen rats were randomly allocated to four groups: control, SPS, DHA, and EPA (*n* = 4 per group). Rats from the control and SPS groups were fed the basal diet, and those from the DHA and EPA groups were fed the diets supplemented with 1.2% DHA/EPA. Daily food intake was measured over 2 weeks by weighing the leftovers. All groups ate comparable amounts (*F*(3, 12) = 2.584, *p* = 0.102; Supporting Information S1: Figure S6). No behavioral or biochemical assays were performed on these pilot animals, and they were not included in the formal experimental dataset.

#### 2.2.3 Formal Experiment of Study 2

The 32 juvenile animals (PND 21) were divided into four groups at random: control, SPS, DHA, and EPA (*n* = 8 per group). All 32 rats completed the experiment without attrition; no animals were excluded from illness or technical reasons. Serum from each rat was split into two equal aliquots for ELISA and non‐targeted lipidomics. For Western blot analysis of the PFC, three rats per group were randomly selected. Groups were fed different diets until adulthood: the control and SPS groups were given a basal diet, whereas the DHA and EPA groups were provided with diets supplemented with 1.2% DHA or 1.2% EPA (Nordic Naturals, Santa Cruz, CA), respectively [37] (see Supporting Information S1: Table S2 for feeding regimens). Weekly diet among the groups is shown in Supporting Information S1: Figure S7. All diets were obtained from Keao Xieli (Beijing) Co. Ltd., and their compositions are detailed in Supporting Information S1: Table S3.

**FIGURE 1:**
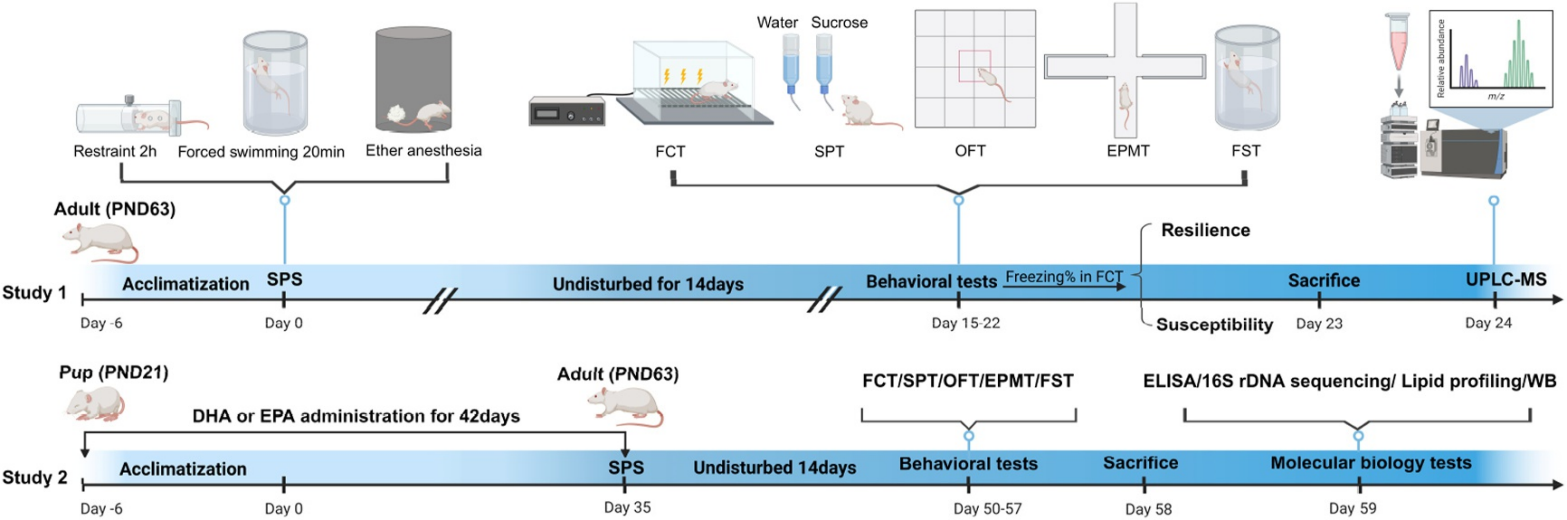
Experimental procedure of this study.

The overall experimental timeline for both studies is illustrated in Figure 1.

#### 2.2.4 Sample Size and Attrition

In Study 1, 48 adult rats were used (control, *n* = 12; SPS, *n* = 36). In Study 2, all 32 rats in the formal experiment completed the behavioral battery and subsequent sample collection without attrition. No animals were excluded due to illness, abnormal behavioral performance, or technical failure. The 16 pilot animals were not included in any statistical analyses reported in this study.

### 2.3 Establishment of Single Prolonged Stress (SPS)

The SPS protocol was performed as previously described [38]. Briefly, rats underwent sequential stressors: (1) 2‐h acute restraint in plexiglas tubes (diameter: 7 cm; length: 20 cm); (2) immediate forced swim for 20 min in cylindrical tanks (height: 46 cm; diameter: 21 cm; water depth: 30 cm; 25 ± 1°C); (3) 15‐min rest followed by exposure to diethyl ether until loss of righting reflex. Finally, the animals were housed in their home cages and remained undisturbed for the subsequent 14 days.

### 2.4 Behavioral Tests

#### 2.4.1 Fear Conditioning Test (FCT)

**FIGURE 2:**
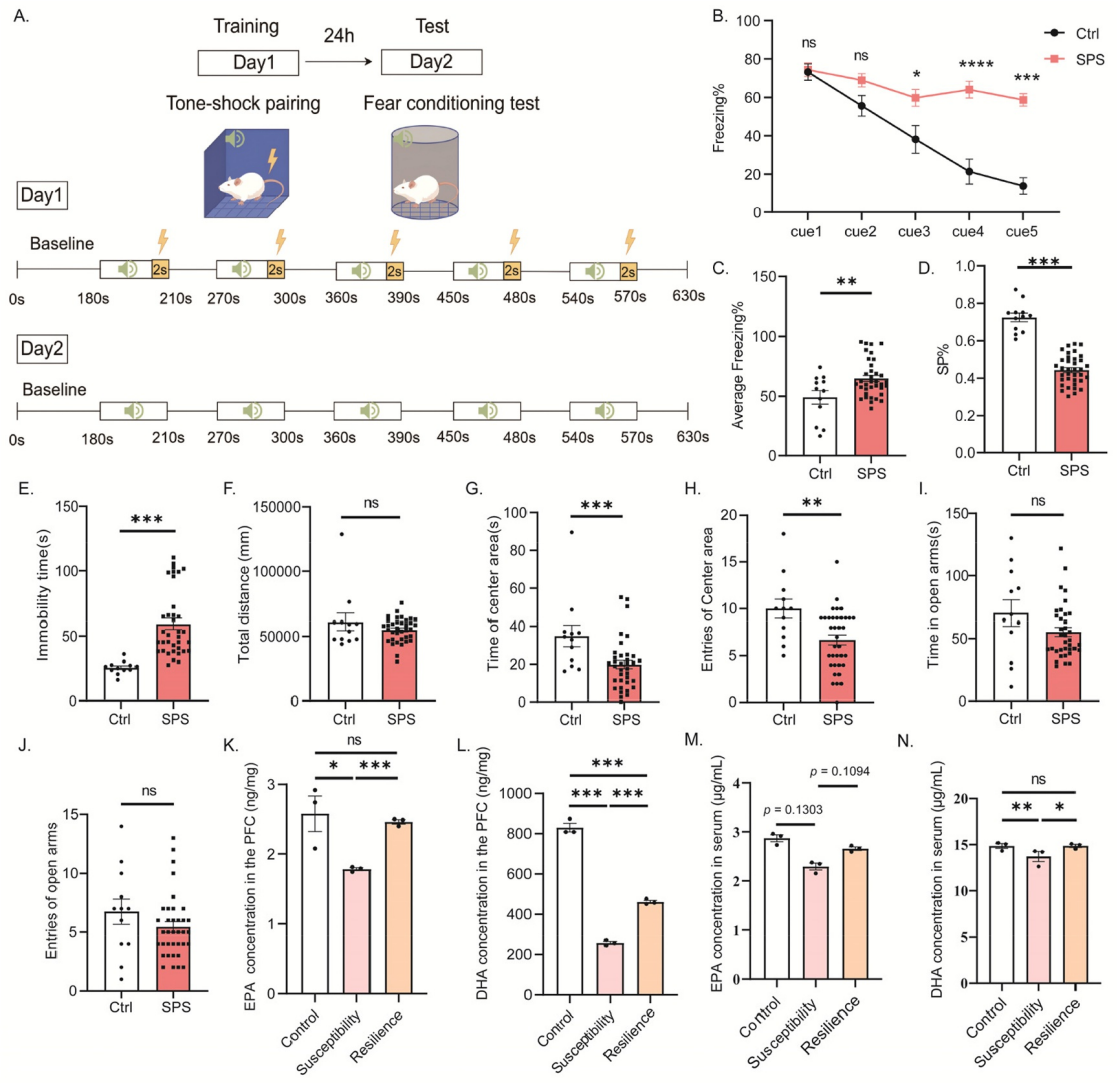
Evaluation of PTSD‐like behaviors and trauma susceptibility/resilience‐associated differences in EPA and DHA levels in the prefrontal cortex (PFC) and serum. (A): Fear conditioning test (FCT) paradigm. (B): Freezing percentage at five cue presentations in FCT. (C): Average freezing percentage throughout FCT. (D): Sucrose consumption in the sucrose preference test (SPT). (E): Immobility time in the forced swim test (FST). (F–H): Total distance, center duration, and center entries in the open field test (OFT). (I, J): Open‐arms time and entries in the elevated plus maze test (EPMT), respectively. (K–N): EPA and DHA concentrations in the PFC and serum across control, susceptibility, and resilience groups evaluated by LC‐MS. For behavioral tests, Ctrl: control group (*n* = 12); SPS: SPS model group (*n* = 36); according to the predefined behavioral classification criterion based on freezing percentage in the FCT, rats within the highest and lowest quartiles were assigned to the susceptible (*n* = 9) and resilient groups (*n* = 9), respectively. Animals with intermediate freezing responses (*n* = 18) were categorized as normal responders and were not included in subsequent analyses. For LC‐MS analysis, prefrontal cortex and serum samples were collected from three randomly selected animals in each of the control, susceptible, and resilient groups (*n* = 3 per group). *p < 0.05, **p < 0.01, ***p < 0.001, ****p < 0.0001, ns, not significant. The same group designations and statistical notations apply to all subsequent figures.

The experiment consisted of two phases. During the training phase (Day 1), rats were introduced into the fear conditioning chamber for 3 min (Segment A), and the freezing time during this period was recorded as the baseline. Freezing behavior, defined as a defensive posture characterized by complete immobility in response to perceived threats [39, 40], was measured. Subsequently, a tone stimulus (80 dB, 30 s) was presented (Segment B), immediately followed by a foot shock (0.5 mA, 2 s) during the last 2 s of the tone (Segment C). The A‐B‐C procedure consisted of five trials. After each training session, the chamber was wiped with 70% ethanol before the next animal was introduced. Twenty‐four hours later (Day 2), we tested fear memory retrieval in a context different from the training environment. During testing, rats were exposed exclusively to the tone cue without foot shock. Freezing responses were quantified automatically using FreezeFrame software (Actimetrics, Evanston, IL), and all sessions were video‐recorded for later documentation and verification (Figure 2A).

#### 2.4.2 Sucrose Preference Test (SPT)

On the first day of the SPT, rats were habituated with two bottles of 1% sucrose solution for 24 h. The next day, they received one bottle of 1% sucrose together with one bottle of plain water. To avoid any side bias, we swapped the bottle positions halfway through. On the third day, after withholding food and water for 22 h [41], we allowed the animals free access to both bottles for 1 hour. We determined how much each rat drank by weighing the bottles before and after the session. Sucrose preference was then calculated as *SP%* = (sucrose intake)/(water intake + sucrose intake) × 100%.

#### 2.4.3 Open Field Test (OFT)

The OFT procedure was based on a previously described method with minor changes [42]. The apparatus consisted of a square chamber (100 × 100 × 40 cm). Each animal was placed in the center of the arena and allowed to move freely for 7 min. Behavioral parameters recorded via an overhead camera from minute 3 to minute 7, including total distance traveled, time spent in the center zone, and number of entries into the center zone. Data were analyzed by AnyMaze software.

#### 2.4.4 Elevated Plus Maze Test (EPMT)

The elevated plus maze apparatus comprises two open arms and two closed arms arranged perpendicularly. Each arm measures 50 cm × 10 cm (length × width). The rats were placed at the central intersection and allowed to explore freely for 5 min [43, 44]. The time spent in and the number of entries into the open arms were recorded by a camera and AnyMaze software.

#### 2.4.5 Forced Swim Test (FST)

Rats were placed in a glass cylinder (21 cm diameter × 46 cm height) containing water (24°C) for 15 min. After 24 h, rats were once again placed in the cylinder for a 5 min period [45]. Following swimming, the animals were warmed for at least 30 min before returning to their home cages. Testing occurred between 11:00 and 15:00. Immobility time during the 5‐min test was recorded. Rats were classified as immobile when they remained floating without active locomotion and displayed only movements sufficient to prevent submersion.

### 2.5 Sample and Tissue Preparation

In Study 1, after all behavioral tests (Day 23), rats were intraperitoneally administered urethane (20% w/v, 1 g/kg). After anesthesia, serum was collected from the abdominal aorta and centrifuged (3000 rpm, 10 min, 4°C). Rats were then rapidly decapitated, and PFC tissue was harvested on ice. The tissue was homogenized on ice in RIPA lysis buffer (Applygen Technology, China) using sonication (30% intensity, 5 s pulses). The homogenate was then centrifuged (12,000 rpm, 15 min, 4°C). All samples were stored at −80°C until subsequent LC‐MS analysis. In Study 2, upon completion of behavioral tests (Day 59), fresh fecal samples were collected by gentle abdominal pressure into sterile tubes and immediately frozen at −80°C for 16S rDNA sequencing. Then, rats were anesthetized as in Study 1. Serum and PFC tissue were collected under identical conditions, with serum split equally into two aliquots for ELISA and lipidomics. PFC tissue was used for Western blot analysis.

### 2.6 Enzyme‐Linked Immunosorbent Assay (ELISA)

The commercial kits (Solarbio Science & Technology, China) were used to quantify serum levels of interleukin‐1 (IL‐1), interleukin‐6 (IL‐6) and tumor necrosis factor‐α (TNF‐α) according to the instructions.

### 2.7 Liquid Chromatography‐Mass Spectrometry (LC‐MS)

Standard solutions were prepared by adding 3 μL of DHA or EPA standard (Sigma‐Aldrich, USA) to 2.826 mL or 2.820 mL of acetonitrile, respectively, yielding individual stock solutions (1000 μg/mL). To obtain a mixed standard solution (40 μg/mL for each analyte), 400 μL of each stock solution was combined with 9.2 mL of acetonitrile. This solution represented the highest calibration standard. Serial dilution with acetonitrile generated calibration standards at concentrations of 40, 20, 10, 5, 2.5, 1.25, 0.625, and 0.3125 μg/mL for both DHA and EPA. A working solution was prepared by diluting 40 μL of the mixed standard (40 μg/mL) with 3960 μL of acetonitrile to a final volume of 4 mL. We performed sample extraction by adding 350 μL of working solution to 25 μL of serum or PFC tissue supernatant. The mixture was briefly vortexed and then centrifuged (13,000 rpm, 5 min, 4°C). We separated DHA and EPA using an ACQUITY UPLC HSS T3 column (100 mm × 2.1 mm, 1.7 μm; Waters Corp., USA). The column was kept at 30°C and connected to a Xevo TQ‐S triple quadrupole mass spectrometer (Waters Corp., USA). For the elution, we applied a gradient of acetonitrile (with 0.1% formic acid) from 40% up to 98% at a flow rate of 0.4 mL/min, and injected 1 μL of each sample. The settings were as follows: capillary voltage of 2.0 kV, source temperature of 150°C, and desolvation temperature of 500°C. Nitrogen (N_2_) was used as the desolvation gas at 600 L/h. Cone voltage and collision energy were configured separately for each compound: 20 V/15 eV for EPA and 20 V/12 eV for DHA. Finally, the data were collected and processed with AB Sciex Analyst and MultiQuant 2.1 software.

### 2.8 16S rDNA Microbial Community Analysis

We extracted genomic DNA using the E.Z.N.A. Stool DNA Kit (Omega Bio‐tek, Norcross, GA, USA). Then, we amplified the V3‐V4 hypervariable regions of the bacterial 16S rRNA gene using primers 338F (5′‐ACTCCTACGGGAGGCAGCAG‐3′) and 806R (5′‐GGACTACHVGGGTWTCTAAT‐3′). An 8‐bp sample‐specific barcode was added to the forward primer, and Illumina adapter sequences were attached to both primers. PCR amplification was performed in a 20 μL reaction system consisting of 0.4 μL FastPfu polymerase, 4 μL FastPfu buffer (5×), 2 μL dNTPs (2.5 mM), 0.8 μL forward and reverse primers (5 μM each), about 15 ng genomic DNA, and nuclease‐free water added to reach the final volume. The PCR program comprised an initial 2‐min denaturation at 95°C, followed by 30 cycles of denaturation (95°C, 30 s), annealing (53°C, 30 s), and extension (72°C, 45 s), with a final extension at 72°C for 10 min. Three technical replicates were performed per sample. Amplicons were visualized following separation on 2% agarose gels. Target amplicons (∼460 bp) were excised from agarose gels, purified with the AxyPrep DNA Gel Extraction Kit (Axygen Biosciences, California, USA), and quantified using QuantiFluor‐ST (Promega, Wisconsin, USA). Samples were normalized, pooled, and sequenced on the Illumina PE250 platform (2 × 250 bp; Illumina, California, USA) by OBiO Technology Corp. Ltd. (Shanghai, China).

### 2.9 Lipid Profiling

Lipidomic profiling was performed using ultra‐performance liquid chromatography coupled to an Orbitrap mass spectrometer. Using LipidSearch software (Thermo Fisher Scientific, Massachusetts, USA), we identified lipids and preprocessed the data. Rat serum sample preparation and mass spectrometric analysis were performed using protocols identical to those described in Section 2.7. Putative lipid identifications were subsequently validated against the LIPID MAPS (https://www.lipidmaps.org) and METLIN (https://metlin.scripps.edu) databases by matching experimental m/*z* values (± 5 ppm) and MS/MS fragmentation patterns.

### 2.10 Western Blot Analysis

PFC samples were lysed in RIPA buffer supplemented with protease and phosphatase inhibitor cocktails (Beyotime Biotechnology, Shanghai, China). Following protein quantification, 50 μg of total protein from each sample was loaded onto SDS‐polyacrylamide gels and electrophoretically separated under stepwise voltage conditions (80 V during stacking and 120 V during separation). Proteins were subsequently transferred onto PVDF membranes (0.45 μm; MilliporeSigma, USA) that had been pretreated with methanol, with a constant current of 400 mA under cooled conditions. The membranes were incubated in TBST containing 5% skimmed milk for 1 h to minimize nonspecific binding, followed by overnight incubation with primary antibodies at 4°C. After three washes with TBST (10 min each), the membranes were exposed to the corresponding HRP‐linked secondary antibodies for 1 h at room temperature. After three final TBST washes, bands were visualized using an ECL detection reagent (Applygen Technologies, Beijing, China) and imaged with a digital imaging system (ProteinSimple, California, USA). Antibody information is listed in Supporting Information S1: Table S4.

### 2.11 Statistical Analysis

Data were analyzed using SPSS Statistics and GraphPad Prism. Figures were generated using BioRender (BioRender.com) and Figdraw (Figdraw.com.cn). Normality was assessed using the Shapiro‐Wilk test. For datasets satisfying assumptions of normality and homogeneity of variance, intergroup differences were analyzed using one‐way analysis of variance (ANOVA), followed by Dunnett's post hoc test for multiple comparisons. When homogeneity of variance was violated, Welch's ANOVA with the Games‐Howell post hoc test was applied. Non‐normally distributed data were analyzed using the Kruskal‐Wallis test with Bonferroni‐corrected pairwise comparisons. Repeated‐measures data were analyzed using repeated‐measures ANOVA, with Mauchly's test used to assess sphericity. Data were presented as mean ± SEM, and statistical significance was defined as *p* < 0.05.

## 3 Results

### 3.1 SPS Induced PTSD‐Like Behaviors

In the FCT, a significant main effect of SPS stress was observed on freezing percentage (Figure 2B, *F*(1, 46) = 22.72, *p* < 0.001) and on time (*F*(4, 184) = 26.26, *p* < 0.001). A significant interaction between stress administration and time was also found (*F*(4, 184) = 10.15, *p* < 0.001). The average freezing percentage across the five cue presentations was significantly increased in SPS‐treated rats (*t*(46) = 2.84, *p* < 0.01, Figure 2C), indicating an enhanced fear response to sound cues. Moreover, SPS administration reduced sucrose preference in the SPT (*t*(46) = 10.75, *p* < 0.001, Figure 2D) and increased immobility time in the FST (*t*(46) = 4.53, *p* < 0.001, Figure 2E), demonstrating depressive‐like phenotypes. There was no difference in the total distance traveled in the OFT (*t*(46) = 1.32, *p* = 0.19, Figure 2F). However, SPS‐treated rats exhibited less time spent in the center (*t*(46) = 2.98, *p* < 0.001, Figure 2G) and fewer entries into the center (*t*(46) = 3.02, *p* < 0.01, Figure 2H), suggesting anxiety‐like behaviors. In the EPMT, however, no significant differences were detected in time spent in the open arms (*t*(46) = 1.72, *p* = 0.092, Figure 2I) or entries into the open arms (*t*(46) = 1.31, *p* = 0.20, Figure 2J). Representative locomotor trajectories from the OFT and EPMT are shown in Supporting Information S1: Figure S1. Overall, these findings indicate that SPS successfully induced PTSD‐like behaviors in rats.

### 3.2 Trauma‐Susceptible Rats Exhibited Reduced DHA and EPA Levels in the PFC and Serum

The 36 SPS model rats were classified into trauma‐resilient, trauma‐susceptible, and normal‐response groups according to their freezing percentage. The behavioral results of these three groups are shown in Supporting Information S1: Figures S2 and S3. We then used LC‐MS to examine whether susceptibility or resilience was associated with differences in EPA and DHA levels in the PFC and serum. In the PFC, the susceptible group showed significantly lower EPA concentrations compared to both control and resilient groups (*t*(4) = 3.09, *p* < 0.05; *t*(4) = 17.82, *p* < 0.001, respectively). A similar pattern was observed for DHA (*t*(4) = 25.13, *p* < 0.001; *t*(4) = 18.94, *p* < 0.001, respectively). Similarly, in serum, DHA concentrations were significantly lower in the susceptible group than in the control and the resilient groups (*t*(4) = 5.82, *p* < 0.01; *t*(4) = 4.59, *p* < 0.05, respectively). However, serum EPA concentrations in the susceptible group only showed a decreasing trend compared to the control and resilient groups (*t*(4) = 1.90, *p* = 0.1303; *t*(4) = 2.05, *p* = 0.1094, respectively) (Figure 2K–N). See Supporting Information S1: Figures S4 and S5 for the chromatograms. Trauma susceptibility was found in this study to be linked to decreased levels of ω‐3 PUFAs. We therefore hypothesize that supplementation with DHA and EPA may help alleviate PTSD‐like symptoms induced by traumatic stress.

### 3.3 DHA and EPA Supplementation in Adolescence Reversed Abnormal Fear Memory Induced by SPS in Adulthood

**FIGURE 3:**
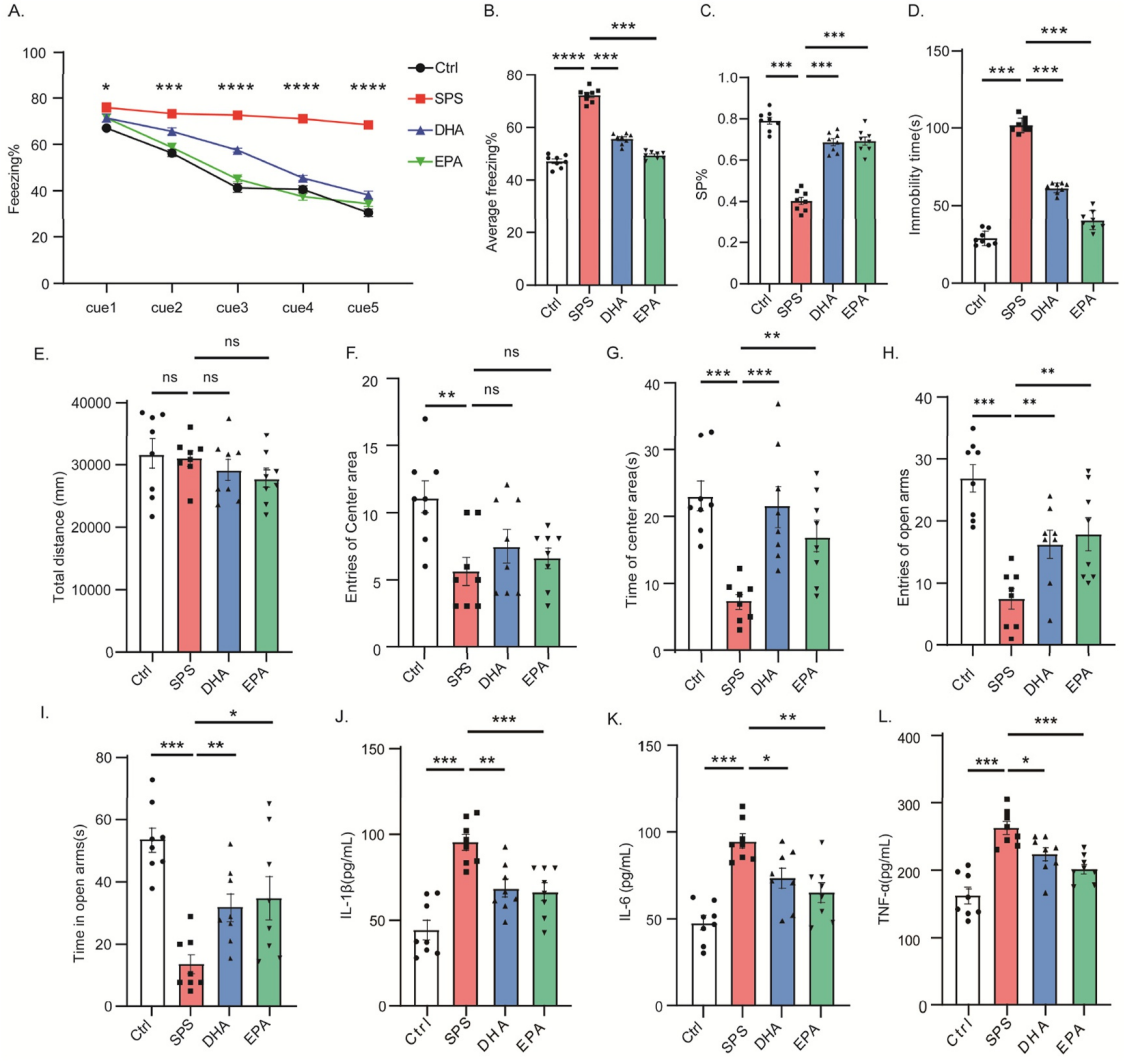
DHA and EPA administration in adolescence alleviated PTSD‐like behaviors induced by SPS in adult rats. (A, B): Freezing percentage at five cue presentations and the average freezing percentage in FCT. (C): The percentage of sucrose consumption in SPT. (D): Immobility time during FST. (E–G): The total distance traveled, number of entries into the center area, and the duration of time spent in the center area during OFT. (H, I): The number of entries and the duration of time spent in the open arms during EPMT. (J–L): Serum levels of IL‐1β, IL‐6, and TNF‐α. For behavioral tests and ELISA analysis of inflammatory cytokines, *n* = 8 per group.

Significant main effects of treatment (Figure 3A,B, *F*(3, 28) = 216.10, *p* < 0.0001) and time (*F*(4, 112) = 223.70, *p* < 0.0001) on freezing percentage were found, as well as a significant treatment × time interaction (*F*(12, 112) = 17.24, *p* < 0.001). The SPS group exhibited heightened fear responses to sound cues (*t*(14) = 19.74, *p* < 0.0001), while the DHA‐ and EPA‐supplemented groups showed a significant improvement in this aberrant fear memory (*t*(14) = 13.87, *p* < 0.001; *t*(14) = 21.34, *p* < 0.001, respectively).

### 3.4 DHA and EPA Supplementation in Adolescence Alleviated Depression‐Like Behavior in Adulthood

DHA and EPA administration altered the sucrose preference in the SPT (Figure 3C, *F*(3, 28) = 87.62, *p* < 0.0001). The reduced SP% in the SPS group was observed (*t*(14) = 15.87, *p* < 0.001). Conversely, both DHA and EPA treatment significantly increased the SP% compared to the SPS group (*t*(14) = 11.70, *p* < 0.001; *t*(14) = 10.90, *p* < 0.001). There was a similar change in immobility time in the FST (Figure 3D, *F*(3, 28) = 355.8, *p* < 0.0001). Significantly more immobility time was observed in the SPS group relative to controls (*t*(14) = 30.80, *p* < 0.001). In contrast, both DHA and EPA administration significantly shortened the immobility time (*t*(14) = 20.45, *p* < 0.001; *t*(14) = 22.50, *p* < 0.001, respectively).

### 3.5 DHA and EPA Supplementation in Adolescence Mitigated Anxiety‐Like Behavior in Adulthood

Meanwhile, both DHA and EPA administration ameliorated anxiety‐like behaviors in adult rats exposed to SPS, as evidenced by their performance in OFT and EPMT. Total locomotor activity in the OFT did not differ between groups (Figure 3E, *F*(3, 28) = 1.042, *p =* 0.40), indicating no general locomotor impairment. A significant main effect of group was found for the number of entries into the center area (Figure 3F, *F*(3, 28) = 5.05, *p <* 0.01). The SPS group entered the center area significantly less often than the control group (*p <* 0.01). This parameter was not significantly improved by either DHA or EPA administration (*p =* 0.26 and *p =* 0.45, respectively). In contrast, the time spent in the center area yielded more pronounced effects (Figure 3G, *F*(3, 28) = 9.65, *p <* 0.001). The SPS group spent significantly less time in the center area than the control group (*p <* 0.001), which was restored by both DHA and EPA treatment (*p <* 0.001 and *p <* 0.01, respectively).

Additionally, in EPMT, significant inter‐group differences were observed in both the number of open arm entries (Figure 3H, *F*(3, 28) = 12.72, *p <* 0.001) and the time spent in the open arms (Figure 3I, *F*(3, 28) = 11.55, *p <* 0.001). Specifically, the SPS group exhibited fewer entries and spent less time in the open arms (*ps* < 0.001), indicating an anxiety‐like phenotype. Both DHA and EPA administration alleviated these deficits, significantly increasing the number of open arm entries (*ps* < 0.01) and the time spent in the open arms (*p* < 0.01 and *p* < 0.05, respectively).

Representative locomotor trajectories from each group in the OFT and EPMT are provided in Supporting Information S1: Figure S8.

### 3.6 Adolescent DHA/EPA Supplementation Reduced Adulthood Serum Inflammatory Factors After SPS

SPS modeling induced a marked increase in all three inflammatory cytokines (Figure 3J–L, IL‐1β: *F*(3, 28) = 16.71, *p <* 0.001; IL‐6: *F*(3, 28) = 15.10, *p <* 0.001; TNF‐α: *F*(3, 28) = 18.94, *p <* 0.001), confirming that SPS elicits a robust inflammatory response. Both DHA and EPA administration reduced the levels of IL‐1β, IL‐6, and TNF‐α (DHA: *p <* 0.01, *p <* 0.05, *p <* 0.05, respectively; EPA: *p <* 0.001, *p <* 0.01, *p <* 0.001, respectively). These results indicate that supplementation with DHA and EPA during adolescence significantly alleviates SPS‐induced inflammatory responses in adulthood.

### 3.7 DHA and EPA Supplementation in Adolescence Correlated With Restoration of Gut Microbial Homeostasis Induced by SPS in Adulthood

#### 3.7.1 Species Composition

**FIGURE 4:**
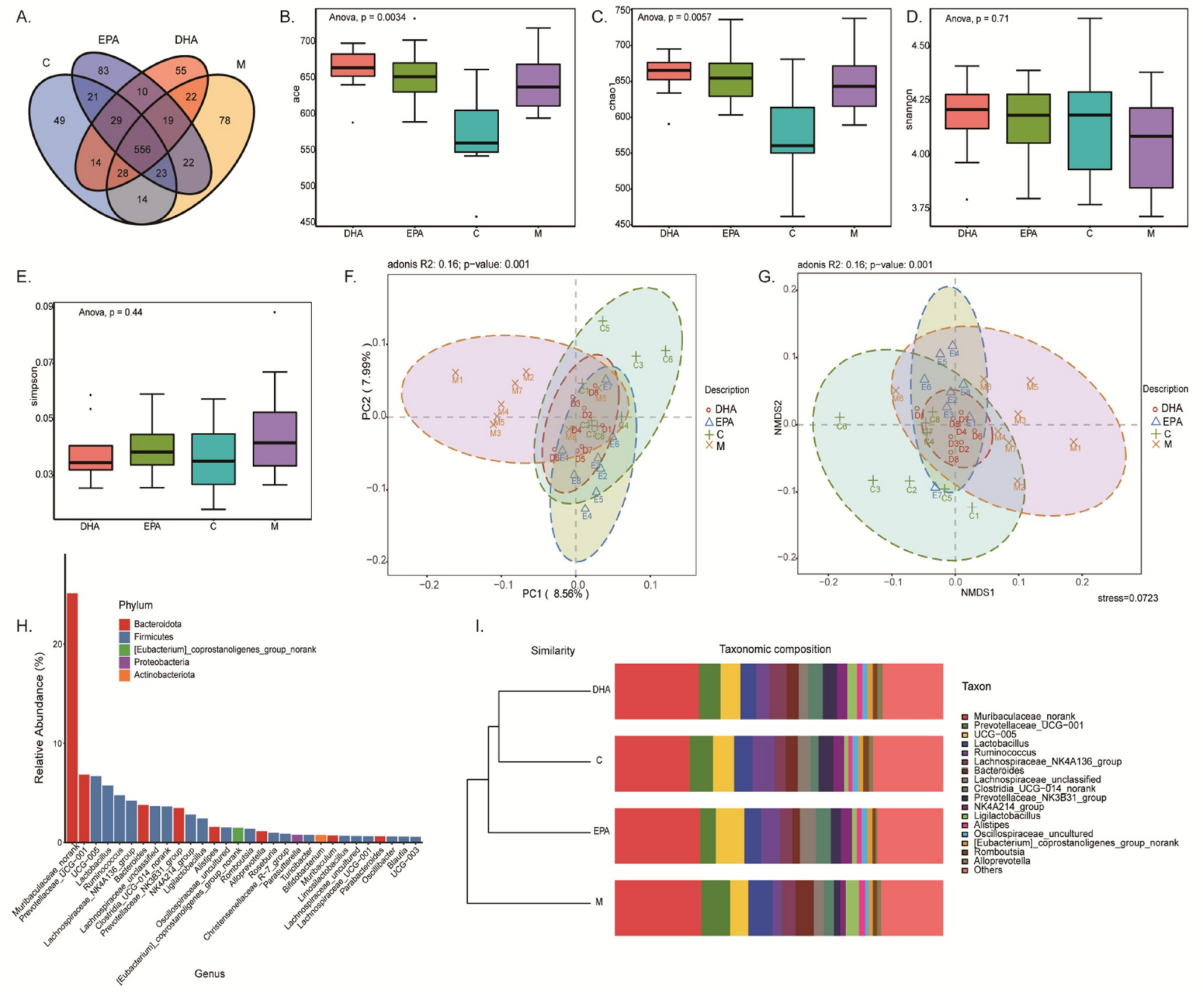
Gut microbiota analysis of fecal samples from each group (*n* = 8). (A): Shared and distinct OTUs identified among groups. (B–E): Alpha diversity indices assessed by the ACE, Chao1, Shannon, and Simpson metrics. (F, G): Beta diversity analyzed by PCoA and NMDS based on the Bray‐Curtis distance. (H): Relative abundance of the dominant microbial taxa. (I): Hierarchical clustering and taxonomic composition analysis of gut microbiota.

Gut symbiotic microorganisms play a broad regulatory role in the stress response [46], suggesting their potential as therapeutic targets for PTSD. We characterized the microbial community composition in rat fecal samples by conducting 16S rDNA gene sequencing. The overlap and count of operational taxonomic units (OTUs) across groups were assessed using Venn diagram analysis. The total numbers of OTUs in the control, SPS, DHA, and EPA groups were 734, 762, 733, and 763, respectively. The numbers of unique OTUs in each group were 49, 78, 55, and 83, with 556 OTUs shared among all four groups (Figure 4A). At the phylum level, the top five species with the highest relative abundance were *Bacteroidota, Firmicutes, Eubacterium‐coprostanoligenes‐group, Proteobacteria* and *Actinobacteriota*. At the genus level, the five most abundant taxa were *Muribaculaceae norank, Prevotellaceae‐UCG‐001, UCG‐005, Lactobacillus* and *Ruminococcus* (Figure 4H).

#### 3.7.2 Gut Microbiota Diversity

Assessment of alpha diversity revealed differences in within‐sample microbial diversity and richness. Significant differences among the groups were detected in the abundance‐based coverage estimator (ACE) (*F*(3, 28) = 5.61, *p* < 0.01, Figure 4B) and the Chao1 index (*F*(3, 28) = 5.05, *p* < 0.01, Figure 4C). The SPS model group showed significant increases in ACE (*p* = 0.022) and Chao1 (*p* = 0.025) values, suggesting that traumatic stress exposure increased gut microbial abundance. However, neither DHA nor EPA supplementation resulted in a significant improvement in ACE or Chao1 values. Moreover, no significant intergroup differences were found in the Shannon and Simpson indices (Figure 4D,E). Beta diversity was characterized by principal coordinate analysis (PCoA) and non‐metric multidimensional scaling (NMDS) to explore inter‐sample differences in microbial community composition. The results demonstrated a clear distinction between the control and model groups. In contrast, the DHA and EPA groups clustered more closely with the control group (Figure 4F,G), indicating that DHA/EPA supplementation ameliorated PTSD‐like dysbiosis. This finding suggests that adolescent supplementation with DHA or EPA ameliorates PTSD‐like dysbiosis of the gut microbiota in adulthood, which is consistent with the results of hierarchical clustering and taxonomic composition analysis (Figure 4I).

#### 3.7.3 Differential Analysis

**FIGURE 5:**
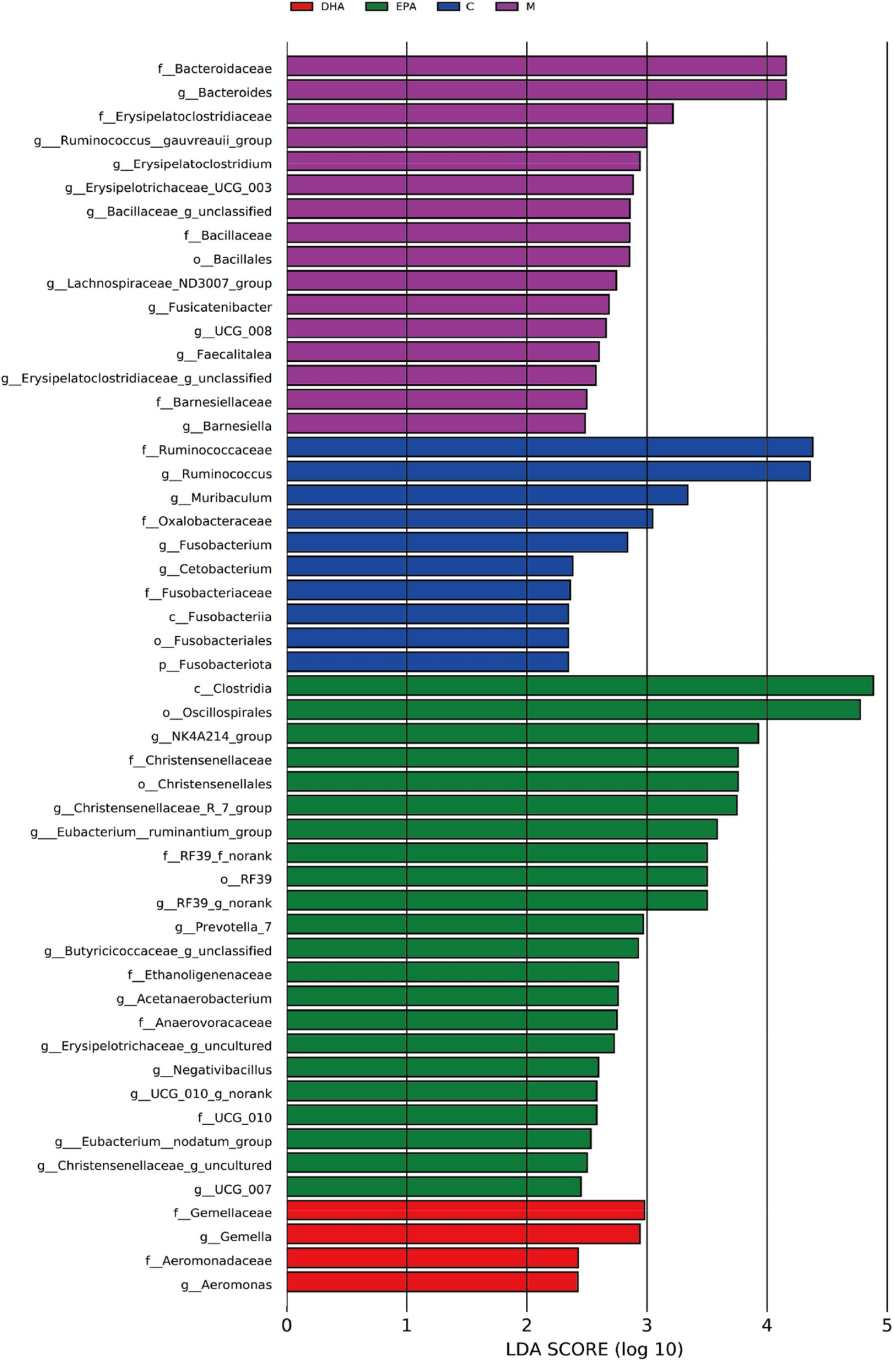
Taxonomic biomarkers of gut microbiota distinguished by LEfSe (LDA score > 4, *p* < 0.05, *n* = 8).

Microbial taxa contributing to group discrimination were determined by linear discriminant analysis effect size (LEfSe) with a threshold LDA score > 4. A higher LDA score reflects a greater contribution of the taxon to intergroup discrimination. Distinct taxonomic signatures were observed across the groups (Figure 5). Ruminococcaceae and *Ruminococcus* were more abundant in the control group, whereas the model group showed higher levels of Bacteroidaceae and *Bacteroides*. EPA treatment was associated with enrichment of *Clostridia* and *Oscillospirales*. In contrast, no microbial features reached the predefined significance threshold (LDA > 4) in the DHA group.

Reduced abundance of Ruminococcaceae and *Ruminococcus* has been reported in conditions characterized by microbial imbalance and metabolic dysfunction [47, 48, 49]. Although members of Bacteroidaceae and *Bacteroides* contribute to the maintenance of intestinal microbial ecology [50], enrichment of specific taxa within these groups has also been associated with pathological conditions, including inflammatory bowel disease [51, 52]. Recent evidence suggests that shifts in the abundance of these microbes relate to stress‐associated disorders, possibly through signaling pathways involved in gut‐brain communication [53]. By contrast, *Clostridia* [54] and *Oscillospirales* [49] are commonly regarded as having anti‐inflammatory properties, partly due to their ability to generate short‐chain fatty acids (SCFAs) [55]. Based on these findings, we hypothesize that Ruminococcaceae and *Ruminococcus* are crucial for maintaining gut microbial homeostasis. Traumatic stress may therefore trigger inflammation by enriching pro‐inflammatory Bacteroidaceae and *Bacteroides*. EPA supplementation appears to mitigate inflammation by potentially enhancing the abundance of beneficial *Clostridia* and *Oscillospirales*. However, DHA may regulate gut microbiota composition in a more global manner rather than selectively enriching or suppressing specific taxa.

### 3.8 DHA and EPA Supplementation in Adolescence Altered Lipid Metabolic Profiles in Adulthood

Gut microbiota dysbiosis is a key mechanism underlying lipid metabolism disorders [29], and changes in lipid profiles may have potential as diagnostic biomarkers for mood disorders [56]. Dietary changes can modulate gut microbiota homeostasis and induce host lipid metabolic reprogramming [57, 58], potentially contributing to PTSD pathophysiology [59]. Polyunsaturated fatty acids participate in the transcriptional regulation of lipid synthesis genes [60]. We therefore hypothesized that variations in the types, distribution, and metabolism of lipids may influence the behavioral effects of DHA and EPA in adolescent rats exposed to traumatic stress. To test this, we performed non‐targeted lipidomics using UPLC‐Orbitrap mass spectrometry.

#### 3.8.1 Lipid Composition

**FIGURE 6:**
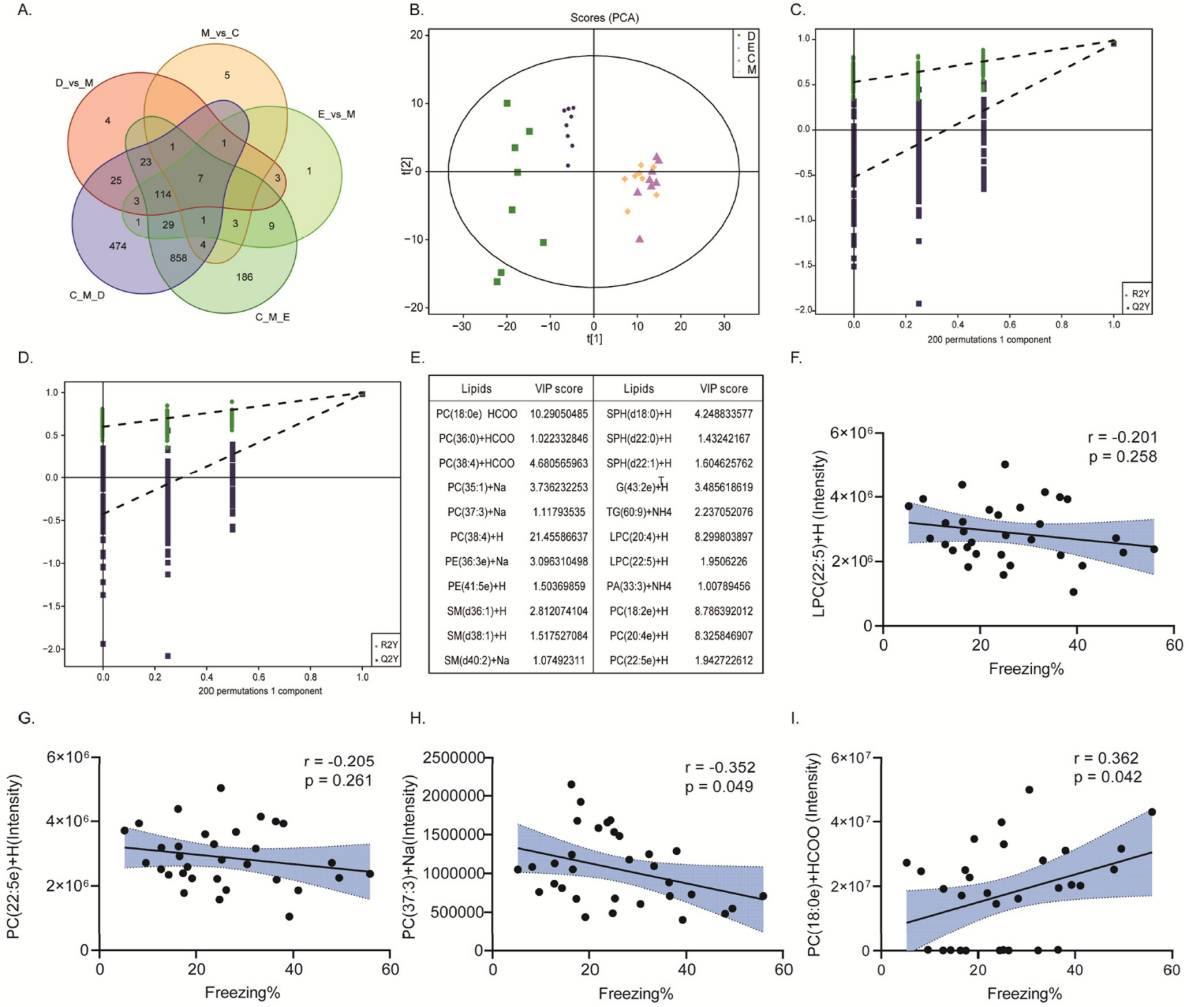
Lipidomics analysis of serum samples from each group (*n* = 8). (A): Distribution of differentially expressed lipids among the groups. (B): Score plot of unsupervised principal component analysis (PCA) (R^2^X = 57.7%). (C, D): OPLS‐DA score plots for DHA versus model and EPA versus model groups, respectively. (E): VIP scores from the OPLS‐DA model. Metabolites with VIP > 1 were considered significant contributors. (F–I): Correlation between freezing percentage in FCT and levels of lysophosphatidylcholine (LPC(22:5) + H) and phosphatidylcholine (PC(22:5e) + H, PC(37:3) + Na, and PC(18:0e) + HCOO). C: control group; M: SPS model group; D: DHA supplementary group; E: EPA supplementary group.

Lipidomic analysis identified 39 lipid classes and 2615 lipid species (Supporting Information S1: Figure S9). Positive and negative ion intensity diagrams of lipid metabolites in serum samples are shown in Supporting Information S1: Figure S10, and the proportions of different lipid compositions are shown in Supporting Information S1: Figure S11. A Venn diagram at the species level reveals both shared and unique lipid molecules across groups (Figure 6A). Unsupervised principal component analysis (PCA) of serum samples showed clear separation among the four groups (R^2^X = 57.7%, Figure 6B). To further distinguish metabolic profiles among groups, pairwise comparisons between the DHA, EPA, and model groups were carried out using orthogonal partial least squares discriminant analysis (OPLS‐DA). Each group exhibited strong clustering with clear separation, indicating distinct lipid profiles. Model reliability was further assessed by cross‐validation ANOVA (CV‐ANOVA; *p* < 0.05) together with 200 permutation tests, both indicating satisfactory performance of the OPLS‐DA model. These results suggest that both DHA and EPA supplementation ameliorate PTSD‐related disturbances in lipid metabolism (DHA vs. model: R^2^Y = 98.8%, *Q* ^2^ = 95.5%; EPA vs. model: R^2^Y = 99.7%, *Q* ^2^ = 97.9%. Figure 6C,D).

#### 3.8.2 Differential Lipid Screening

Differential lipid species were defined by variable importance in projection (VIP) > 1 and FDR‐adjusted *p*‐value (*q*‐value) < 0.05 (Figure 6E). Notably, the levels of lysophosphatidylcholine (LPC(22:5) + H) and phosphatidylcholine (PC(22:5e) + H, PC(37:3) + Na, and PC(18:0e) + HCOO) showed different patterns across the groups. Correlation analysis was further performed between these lipid levels and the freezing percentage in the FCT, a core behavioral phenotype of PTSD (Figure 6F–I). Results from *Pearson's* correlation showed that the freezing percentage was significantly negatively correlated with PC(37:3) + Na and positively correlated with PC(18:0e) + HCOO. These results point to a connection between PTSD and abnormal lipid metabolism, characterized by decreased PC(37:3) + Na and increased PC(18:0e) + HCOO, highlighting a close relationship between phosphatidylcholine and PTSD.

### 3.9 DHA and EPA Supplementation Was Associated With Restoration of PI3K/AKT/mTOR Signaling in PTSD‐Like Rats

We found that phosphatidylcholine levels were significantly correlated with freezing behavior in PTSD‐like rats. Phosphatidylcholine, a major phospholipid constituent of cell membranes, acts as a precursor for the synthesis of lipid signaling molecules. The phosphatidic acid generated through phospholipase D2‐mediated catabolism of phosphatidylcholine functions as a pivotal activator of the phosphatidylinositol 3‐kinase (PI3K)/AKT/mTOR survival signaling cascade [61, 62]. We therefore postulate that the PI3K/AKT/mTOR pathway may be implicated in fear memory dysregulation in PTSD.

**FIGURE 7:**
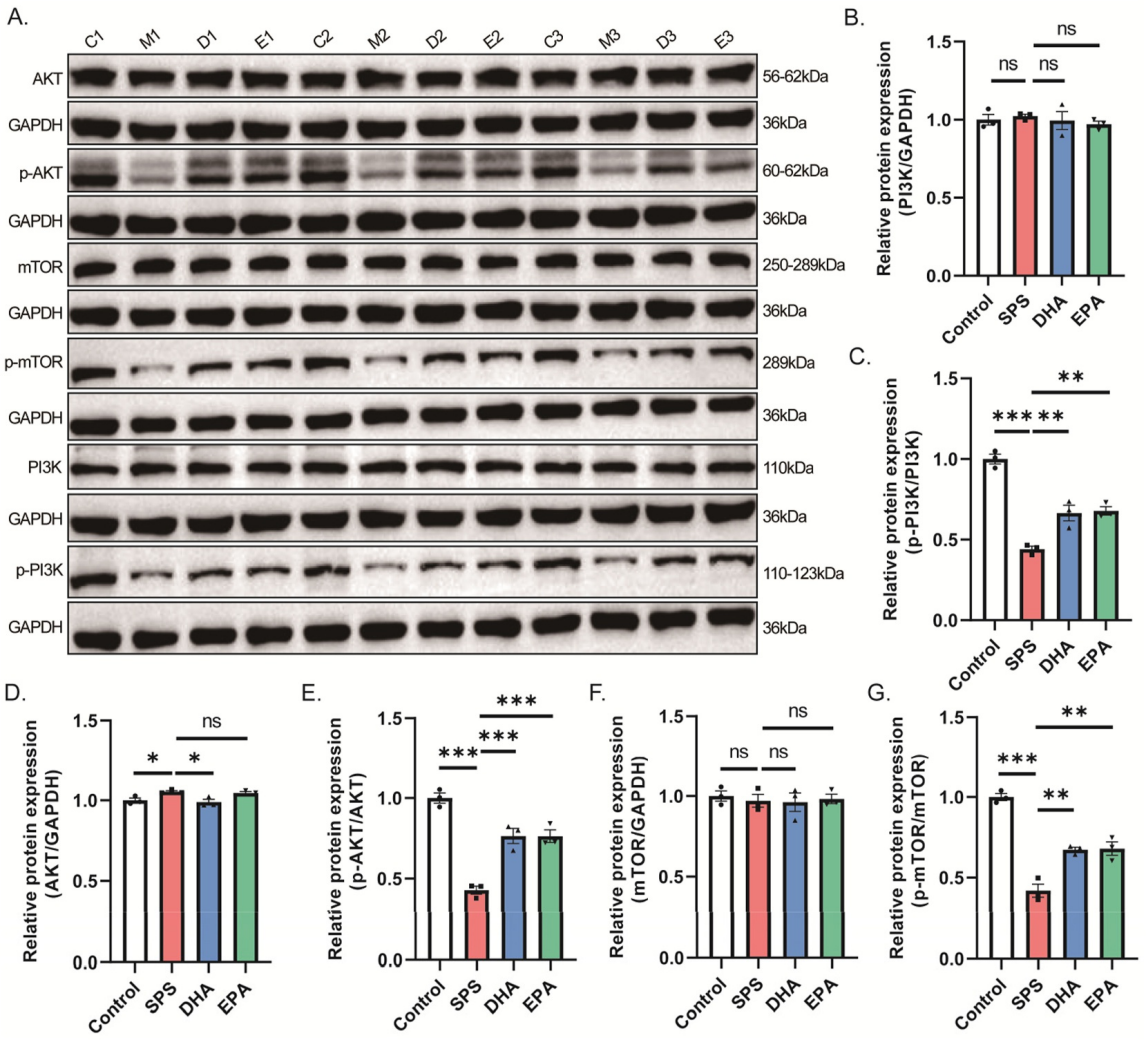
Expression of the prefrontal PI3K/AKT/mTOR pathway in SPS‐induced PTSD model rats after dietary supplementation with DHA and EPA (*n* = 3). (A): Immunoblots of PI3K, phospho‐PI3K, AKT, phospho‐AKT, mTOR, phospho‐mTOR, and GAPDH. (B–G): The quantitative analyses of the protein levels of PI3K/GAPDH, p‐PI3K/PI3K, AKT/GAPDH, *p*‐AKT/AKT, mTOR/GAPDH, *p*‐mTOR/mTOR ratio.

Significant differences in total AKT protein expression were detected among groups via Western blotting (*F*(3, 8) = 6.772, *p* < 0.05), but not in PI3K (*F*(3, 8) = 0.355, *p* = 0.787) or mTOR (*F*(3, 8) = 0.161, *p* = 0.920). We observed that total AKT levels were significantly increased in the SPS group compared to the control animals (*p* < 0.05), which was restored by DHA supplementation (*p* < 0.05) but not by EPA (*p* = 0.40). However, pronounced group differences were observed in the phosphorylation levels of all three proteins (p‐PI3K: *F*(3, 8) = 50.34, *p* < 0.001; *p*‐AKT: *F*(3, 8) = 42.16, *p* < 0.001; *p*‐mTOR: *F*(3, 8) = 53.31, *p* < 0.001). Specifically, the SPS group exhibited markedly decreased p‐PI3K, *p*‐AKT, and *p*‐mTOR levels compared to controls (*ps* < 0.001). Both DHA and EPA supplementation significantly increased the p‐PI3K (SPS vs. DHA: *p* < 0.01; SPS vs. EPA: *p* < 0.01), *p*‐AKT (SPS vs. DHA: *p* < 0.001; SPS vs. EPA: *p* < 0.001), and *p*‐mTOR (SPS vs. DHA: *p* < 0.01; SPS vs. EPA: *p* < 0.01) levels compared to the SPS group (Figure 7A–G). These results indicate that PTSD‐like rats induced by SPS exhibit reduced activation of the prefrontal PI3K/AKT/mTOR pathway, and supplementation with either DHA or EPA reverses this impairment.

## 4 Discussion

This study found that traumatic susceptibility was accompanied by reduced DHA and EPA levels in the PFC. Importantly, adolescent dietary supplementation with DHA or EPA conferred resilience to later traumatic stress and to attenuated SPS‐induced PTSD‐like phenotypes in adulthood, including excessive fear memory, depressive‐ and anxiety‐like behaviors. These behavioral improvements were associated with restoration of gut microbiota homeostasis, lipid metabolic reprogramming, and normalization of PI3K/AKT/mTOR signaling activity in the PFC.

Earlier work demonstrated that omega‐3 administration ameliorates PTSD‐related memory deficits in adult rodents and primarily attributed these effects to oxidative stress regulation [24, 63]. Clinical and epidemiological evidence has further suggested that higher ω‐3 PUFA status may be associated with reduced PTSD risk and improved stress adaptation [23]. More recently, longitudinal human studies linked circulating unsaturated fatty acids with PTSD symptom severity [25]. However, previous studies have rarely considered developmental timing as a determinant of treatment responsiveness. Given that adolescence represents a sensitive period for maturation of prefrontal‐limbic circuits and long‐term stress programming [7, 8, 9, 10, 64, 65], the present study shifts the focus from adult intervention to adolescent nutritional programming and demonstrates that early‐life DHA/EPA exposure may confer long‐lasting resilience against both fear‐related and affective manifestations of PTSD‐like behaviors. Furthermore, by integrating behavioral assessment with microbiome, lipidomic, inflammatory, and signaling analyses, our findings provide preliminary evidence for biological processes that may mediate the effects of ω‐3 PUFAs on susceptibility to traumatic stress.

Recent research on traumatic susceptibility and resilience has provided new insights into early intervention strategies of PTSD. Although exposure to traumatic events typically triggers acute stress responses, only a subset of individuals (susceptible individuals) eventually develop PTSD, while others recover naturally after the initial reaction [66]. It is also noteworthy that certain regions of the PFC continue to develop beyond early childhood [67]. The structure and function of the PFC are associated with fear processing [68]. Distinct patterns of neuronal morphology in the PFC have been reported between mice displaying susceptibility or resilience to PTSD‐like behaviors [69]. Clinical studies have also reported altered dynamic functional connectivity within the PFC in individuals with PTSD, which aligns with the observations described above [70].

There is growing recognition that ω‐3 PUFAs help regulate biological responses to stress. Several studies have tied DHA and EPA to a range of functions: neuroimmune regulation [71, 72], endocannabinoid signaling [73], synaptic remodeling [74], and HPA axis activity [75]. Even so, little is known about how ω‐3 PUFAs might directly contribute to PTSD. Here we used the SPS paradigm to model PTSD‐like phenotypes in rats, which showed enhanced fear responses along with depression‐ and anxiety‐like behaviors. When we measured lipid levels by LC‐MS, susceptible animals were found to have lower DHA and EPA concentrations in the PFC than either control or resilient rats. This points to a possible link between altered lipid metabolism in a stress‐sensitive brain region and the mechanisms that support resilience. It also raises the possibility that when DHA and EPA are in short supply, an individual may become more vulnerable after trauma. Conversely, adolescent supplementation with DHA and EPA conferred neuroprotection. Specifically, supplemented adolescent rats showed reduced freezing behavior and attenuated fear generalization in adulthood when exposed to trauma‐related cues. DHA and EPA supplementation ameliorated depression‐like behaviors, including anhedonia and behavioral despair, and alleviated anxiety‐like symptoms in adult rats. Collectively, adolescent supplementation with DHA and EPA mitigates SPS‐induced PTSD‐like fear memory along with depressive and anxiety‐like behaviors in adulthood.

Diet modulates neurological physiology via the gut‐brain axis, with the gut microbiota acting as a critical mediator. Nutritional interventions during developmental periods can exert long‐term programming effects on microbial composition, immune function, and neural development [76, 77]. The large‐scale data generated from metabolomics and microbiome studies offer a global perspective [78, 79], thus providing valuable insights into the current molecular framework of PTSD. In this study, dietary supplementation with DHA and EPA was provided during the critical window of adolescence. We found that although both fatty acids modulated the abundance and composition of the gut microbiota, they exerted distinct regulatory effects in PTSD‐like rats. Enrichment of Bacteroidaceae and *Bacteroides* was evident in PTSD rats, which may indicate a shift in microbial composition associated with disease state [80]. In contrast, EPA supplementation was associated with increased abundance of *Clostridia* and *Oscillospirales*. The LEfSe analysis showed that DHA treatment did not produce any clearly discriminative taxa, as no features reached the LDA threshold of 4. Thus, rather than a few specific bacteria driving the change, it appears to be a relatively widespread general shift in the gut microbial community.

PTSD involves breakdown in metabolism, inflammation, and gut microbiome [81, 82, 83]. In our study, alterations in lipid metabolism, particularly phosphatidylcholine (PC), stood out as a key metabolic signature of PTSD‐like phenotypes. PC is the most abundant phospholipid in mammalian cells, making up about 40%–50% of cellular phospholipids [84]. It also contributes to acetylcholine synthesis [85, 86], which implies a correlation with the cholinergic changes seen in PTSD [87]. Moreover, PC acts as a fuel for gut microbes, and its own metabolism depends on microbial activity [88]. Stress exposure is known to disrupt intestinal barrier integrity and the microbial balance [89], so this dysfunction can in turn affect systemic lipid metabolism, including pathways involving PC [90]. These types of gut‐lipid interactions are thought to influence brain function indirectly, via inflammatory signaling and microbial byproducts such as short‐chain fatty acids [91].

We found that PC levels correlated with PTSD‐like freezing behavior, but acted in different ways. Specifically, PC(37:3) + Na had a negative association with freezing, while PC(18:0e) + HCOO showed a positive association. This suggests that different PC molecular species may have distinct functions when it comes to stress‐related behaviors.

Given that PC changes correlate with PTSD‐like fear memory, and phospholipid signaling can influence the PI3K/AKT/mTOR pathway, we propose that this pathway may be associated with the effects of ω‐3 PUFA supplementation in PTSD. PI3K activates and orchestrates AKT and mTOR, which in turn promote synaptogenesis [92], reduces neuroinflammation [93], and establishes molecular foundations for emotional and behavioral regulation after trauma exposure. Our study further implicates the PI3K/AKT/mTOR pathway in the pathophysiological processes of PTSD. SPS exposure led to a marked decrease in the phosphorylation levels of PI3K, AKT, and mTOR. Notably, chronic dietary supplementation with DHA and EPA during adolescence effectively reversed these alterations, restoring PI3K/AKT/mTOR pathway activity. In summary, these results suggest that modulation of prefrontal PI3K/AKT/mTOR signaling may exert the beneficial effects of DHA and EPA on PTSD‐related fear memory and affective dysregulation.

While these findings are promising, several limitations should be acknowledged. First, we only used male rats in this study, which helped reduce biological variability and made it easier to interpret the long‐term effects of adolescent ω‐3 PUFA supplementation. However, it also limits the generalizability of our findings to females. This consideration is particularly important because some evidence indicates that PTSD exhibits pronounced sex differences in prevalence. Generally, females show greater susceptibility following trauma exposure [94, 95, 96]. Also, sex may influence gut microbiota composition, lipid metabolism, and neural responses to stress [97, 98]. Thus, the microbiota‐lipid metabolism‐PI3K/AKT/mTOR axis identified here may not be entirely identical in female animals. Future studies should include both sexes to clarify whether the observed protective effects of adolescent DHA/EPA supplementation differ by sex.

Additionally, while the present findings revealed concurrent changes in gut microbial profiles, lipid metabolism, and PI3K/AKT/mTOR signaling following DHA/EPA supplementation, the interactions among these have not yet been fully clarified. Exploring the effects of microbiota‐targeted interventions, such as antibiotic treatment or fecal microbiota transplantation, and pharmacological inhibition of the PI3K/AKT/mTOR pathway will be important for elucidating the causal mechanisms.

## 5 Conclusion

This study provides the first evidence of a significant association between PTSD susceptibility and reduced levels of ω‐3 PUFAs in the PFC and serum. Taking adolescence as the key intervention period, we found that dietary DHA and EPA supplementation attenuated SPS‐induced fear memory as well as depressive‐ and anxiety‐like behaviors in adulthood. The behavioral effects were accompanied by alterations in gut microbial composition and lipid metabolic profiles. Notably, phosphatidylcholine metabolism was linked to freezing behavior in PTSD‐modeled rats, although PC(37:3) + Na and PC(18:0e) + HCOO exhibited opposite patterns of association. The observed changes in PI3K/AKT/mTOR signaling further suggest that this pathway may contribute to the beneficial effects of adolescent ω‐3 PUFA supplementation on stress resilience.

## Author Contributions

Jianyou Guo designed this work. Yi Yin, Wenjing Li, Jie Li, and Ziwei Yao conducted the experiments. Wenjing Li and Yi Yin performed the data analysis. Siqi Wang and Yinan Jin prepared the sample and analyzed the results. Wenjing Li and Yi Yin wrote the manuscript. Lihua Bian gave advice on the manuscript. Jianyou Guo supervised the research and edited the paper.

## Ethics Statement

Experimental procedures complied with the *Guide for the Care and Use of Laboratory Animals* (National Institutes of Health) and were approved by the Ethics Committee of Institute of Psychology, Chinese Academy of Sciences (No. A22067).

## Conflicts of Interest

The authors declare no conflicts of interest.

## Supporting information


Supporting Information S1

## Data Availability

The data that support the findings of this study are available from the corresponding author upon reasonable request.
