# Supplementary material for: Adolescent Dietary Supplementation of Omega‐3 Polyunsaturated Fatty Acids Alleviates PTSD‐Like Fear Memory and Affective Dysregulation in Rats
Source: Smart Med. 2026 Aug 4;5(4):e70049. doi: 10.1002/smmd.70049 (PMC13459811; doi:10.1002/smmd.70049)
Supplement: Supplementary file 1 [file SMMD-5-e70049-s001.doc]

Supplementary material

Table S1. Animal allocation and sample sizes used in each study

|  | **Grouping** | **Sub-grouping** | **Experiments** |
| --- | --- | --- | --- |
| **Study 1 (n = 48)** | Control group (n = 12) | _ | (1)Behavioral tests: all animals from the Control and SPS groups (n = 48);  (2)LC-MS: randomly selected three animals from the Control/Resilience/Susceptibility group (n = 3 per group) |
|  | SPS group (n = 36) | Resilience group (n = 9) |  |
|  |  | Normal response group (n = 18) |  |
|  |  | Susceptibility group (n = 9) |  |
| **Study 2 (*n* = 48)** | Pilot experiment (n = 16) | Control group (n = 4) | Dietary palatability assessment |
|  |  | SPS group (n = 4) |  |
|  |  | DHA group (n = 4) |  |
|  |  | EPA group (n = 4) |  |
|  | Formal experiment (n = 32) | Control group (n = 8) | (1)Behavioral tests/ELISA/16S rDNA/Lipid profiling: all animals from the Control/SPS/DHA/EPA groups (n = 8 per group);  (2)Western Blot: randomly selected three animals from the Control/SPS/DHA/EPA group (n = 3 per group) |
|  |  | SPS group (n = 8) |  |
|  |  | DHA group (n = 8) |  |
|  |  | EPA group (n = 8) |  |

**Table S2.** **Feeding regimens of each group in formal experiment of Study 2**

| Group | Animal Number | Feeding regimens | SPS procedure |
| --- | --- | --- | --- |
| Control group | 8 | standard chow | − |
| SPS group | 8 | standard chow | + |
| DHA group | 8 | DHA chow | + |
| EPA group | 8 | EPA chow | + |

**Table S3. Diet composition**

| Ingredient | Control/SPS groups(g/kg) | DHA group(g/kg) | EPA group(g/kg) |
| --- | --- | --- | --- |
| Corn starch | 425.6 | 413.3 | 413.3 |
| Casein | 220 | 220 | 220 |
| Saccharose | 212.8 | 212.8 | 212.8 |
| Cellulose | 20 | 20 | 20 |
| Mineral mixtur | 40 | 40 | 40 |
| Vitamin mixture | 10 | 10 | 10 |
| DL Methionine | 1.6 | 1.6 | 1.6 |
| Peanut oil | 48.1 | 48.1 | 48.1 |
| Rapeseed oil | 21.9 | 21.9 | 21.9 |
| DHA | − | 12 | − |
| EPA | − | − | 12 |
| Total | 1000 | 1000 | 1000 |

**Table S4. The information of antibodies for Western Blot**

| Antibody | Supplier | Catalog Number | Dilution |
| --- | --- | --- | --- |
| anti-PI3K | Proteintech | 67071-1-Ig | 1:1000 |
| anti-phospho-PI3K | Bioss | Bs-6417R | 1:1000 |
| anti-Akt | Proteintech | 10176-2-AP | 1:1000 |
| anti-phospho-Akt | Proteintech | 66444-1-Ig | 1:1000 |
| anti-mTOR | Proteintech | 66888-1-Ig | 1:1000 |
| anti-phospho-mTOR | Proteintech | 67778-1-Ig | 1:1000 |
| anti-GAPDH | Proteintech | 60004-1-Ig | 1:1000 |
| Goat anti-Rabbit | Beyotime | A0208 | 1:4000 |

**Figure S1.** The representative locomotor trajectories of the control group and the single prolonged stress (SPS) group in the open field test and elevated plus maze test in Study 1. SPS: single prolonged stress group.

**Figure S2.** PTSD-like behaviors induced by SPS in the control group (*n* = 12), trauma-susceptible group (*n* = 9), and trauma-resilient group (*n* = 9) in Study 1. (A). Average freezing percentage in the fear conditioning test. (B). Sucrose consumption percentage in the sucrose preference test. (C). Immobility time in the forced swim test. (D-F). Total distance, entries into the center area, and time spent in the center area of the open field test. (G-H). Entries into the open arms and time spent in the open arms of the elevated plus maze test. All the data are presented as mean ± SEM. Significant differences: * *p* < 0.05, ** *p* < 0.01, *** *p* < 0.001, *ns* = not significant. Ctrl: control group.

**Figure S3.** The representative locomotor trajectories of the control group, trauma-susceptible group, and trauma-resilient group in the open field test and elevated plus maze test in Study 1.

**Figure S4.** Secondary mass spectrum fragment ion diagrams of DHA and EPA in serum. (A): the MS/MS fragment ion spectrum characterizing DHA; (B): the quantitative MS/MS fragment ion profile for DHA; (C): the MS/MS fragment ion spectrum characterizing EPA; (D): the quantitative MS/MS fragment ion profile for EPA.

**Figure S5.** Total ion chromatogram (TIC) in liquid chromatography-tandem mass spectrometry (LC-MS/MS) analysis of the prefrontal cortex extract. A: TIC for the DHA standard; B: TIC for the EPA standard; C-D: Chromatogram (m/z 329.2475 and m/z 303.2300) of the prefrontal cortex extract from the control group; E-F: Chromatogram (m/z 329.2475 and m/z 303.2300) of the prefrontal cortex extract from the trauma-resilient group; G-H: Chromatogram (m/z 329.2475 and m/z 303.2300) of the prefrontal cortex extract from the trauma-susceptible group.

**Figure S6.** Average daily food intake during two weeks in the Control, SPS, DHA, and EPA group in the pilot experiment of Study 2 (*n* = 4). Sixteen juvenile rats (*n* = 4 per group) were fed either the basal diet (the Control and SPS groups) or the 1.2% DHA/EPA-supplemented diets (the DHA/EPA groups) for 2 weeks. Daily food intake was monitored by weighing the remaining feed, and daily averages were calculated. No significant differences in food intake were observed among the four groups (*F*(3, 12) = 2.584, *p* = 0.102). Data are presented as mean ± SEM. *ns*, not significant.

**Figure S7.** Average weekly food intake (g) in Control, SPS, DHA, and EPA Group from acclimation to sacrifice in the formal experiment of Study 2 (*n* = 8). No significant differences in food intake were detected among the Control, SPS, DHA, and EPA groups during weeks 1-6 (*ps* > 0.05). This pattern changed following SPS model induction at postnatal day (PND) 63. Specifically, the SPS group exhibited significantly lower food intake than the other three groups during weeks 7, 8, and 9 (week 7: *F*(3, 28) = 73.63, *p* < 0.0001; Control vs. SPS: *p* < 0.0001; Control vs. DHA: *p* < 0.0001; Control vs. EPA: *p* < 0.05; SPS vs. DHA: *p* < 0.0001; SPS vs. EPA: *p* < 0.0001; DHA vs. EPA: *p* < 0.01. Week 8: *F*(3, 28) = 193.4, *p* < 0.0001; Control vs. SPS: *p* < 0.0001; Control vs. DHA: *p* = 0.45; Control vs. EPA: *p* < 0.05; SPS vs. DHA: *p* < 0.0001; SPS vs. EPA: *p* < 0.0001; DHA vs. EPA: *p* = 0.25. Week 9: *F*(3, 28) = 109.8, *p* < 0.0001; Control vs. SPS: *p* < 0.0001; Control vs. DHA: *p* = 0.59; Control vs. EPA: *p* = 0.99; SPS vs. DHA: *p* < 0.0001; SPS vs. EPA: *p* < 0.0001; DHA vs. EPA: *p* = 0.74). *****p* < 0.0001; SPS: single prolonged stress group; DHA: DHA supplementation group; EPA: EPA supplementation group.

**Figure S8.** The representative locomotor trajectories of the control group, single prolonged stress (SPS) group, DHA supplementation group, and EPA supplementation group in the open field test and elevated plus maze test in the formal experiment of Study 2. SPS: single prolonged stress group; DHA: DHA supplementation group; EPA: EPA supplementation group.

**Figure S9**. Identification and classification of lipid metabolites in serum samples.

*Note.* AcCa: Acyl Carnitine; Cer: Ceramides; CerG2GNAc1: N-acetylhexosyl ceramide; CerG3GNAc1: Dihexosyl N-acetylhexosyl ceramide; CerP: Ceramides phosphate; ChE: Cholesterol Ester; CL: Cardiolipin; Co: Coenzyme; DG: diglyceride; FA: Fatty Acyls; GD2: Ganglioside, disialo dihexosyl ceramide; GM3: Ganglioside, monosialo trihexosyl ceramide; Hex1Cer, Hex2Cer and Hex3Cer: Hexosyl ceramide; LPC: lysophosphatidylcholine; LPE: lysophosphatidylethanolamine; LPI: lysophosphatidylinositol; LPS: lysophosphatidylserine; MG: monoglyceride; OAHFA: (O-acyl)-1-hydroxy fatty acid; PA: phosphatidic acid; PC: phosphatidylcholine; PE: phosphatidylethanolamine; PG: phosphatidylglycerol; phSM: phytosphingosine; PI: phosphatidylinositol; PIP: phosphatidylinositol(4)phosphate; PIP2: phosphatidylinositol(4,5)bisphosphate; PIP3: phosphatidylinositol(3,4,5)triphosphate; PS: phosphatidylserine; SiE: Sitosterol ester; SM: sphingomyelin; SPH: Sphingosine phosphate; ST: Sulfatide; StE: Stigmasterol ester; TG: triglyceride; WE: wax exters; ZyE: zymosterol.

**Figure S10**. Positive and negative ionic strength diagram of lipid metabolites in serum samples.

**Figure S11**. Proportion of lipid components in serum samples.

*Note.* C: control group; M: SPS model group; D: DHA administration group; E: EPA administration group. The abbreviated nomenclature employed for lipid classification in Figure S11 maintains consistency with the conventions previously established in Figure S9.
